# Preparation of Biocolorant and Eco-Dyeing Derived from Polyphenols Based on Laccase-Catalyzed Oxidative Polymerization

**DOI:** 10.3390/polym10020196

**Published:** 2018-02-15

**Authors:** Fubang Wang, Jixian Gong, Xinqing Zhang, Yanfei Ren, Jianfei Zhang

**Affiliations:** 1 School of Textiles, Tianjin Polytechnic University, Tianjin 300387, China; tjpuwfb13@163.com (F.W.); gongjixian@126.com (J.G.); jeanking5056@hotmail.com (X.Z.); tjpuryf@126.com (Y.R.); 2 Key Laboratory for Advanced Textile Composites of the Education Ministry of China, Tianjin 300387, China

**Keywords:** biocolorant, laccase, dyeing, pH value, oxidative polymerization

## Abstract

Natural products have been believed to be a promising source to obtain ecological dyes and pigments. Plant polyphenol is a kind of significant natural compound, and tea provides a rich source of polyphenols. In this study, biocolorant derived from phenolic compounds was generated based on laccase-catalyzed oxidative polymerization, and eco-dyeing of silk and wool fabrics with pigments derived from tea was investigated under the influence of pH variation. This work demonstrated that the dyeing property was better under acidic conditions compared to alkalinity, and fixation rate was the best when pH value was 3. Furthermore, breaking strength of dyed fabrics sharply reduced under the condition of pH 11. Eventually, the dyeing method was an eco-friendly process, which was based on bioconversion, and no mordant was added during the process of dyeing.

## 1. Introduction

With the consumer’s enhanced awareness of eco-safety, widespread interest has emerged in the application of sustainable and eco-friendly materials [1,2,3]. In the textile industry, a constantly increasing interest in biomass pigments has been aroused in recent years [4,5,6], which has been regarded as an ecological, as well as sustainable dyeing technology to address environmental contamination issues caused by the application of synthetic dyestuffs [6,7,8,9,10].

With the ever-increasing demand of biomass colorants [11], several methods have been made to prepare biomass dyestuffs biologically, and to further enhance the content of pigments over the past few years [12,13,14,15]. The most promising is the application of enzyme generated by microorganism to synthesize biopigment [16,17,18]. Biosynthesis is green and secure compared to chemical synthesis [19,20,21,22], which could give rise to effective preparation for target product via biotransformation [12,23].

Considering safety, energy, and water conservation, as well as environmental responsibility, enzymes are gaining an increasing role in textile wet processing [24], and the textile industry has become one of the main fields for industrial application of enzymes [25,26]. Moreover, new enzymes are being introduced to the field of textile processing [27,28]. 

Laccase is regarded as an ideal biocatalyst to take the place of chemical catalysts, by virtue of numerous strengths [29,30], such as high catalytic efficiency, mild reaction conditions, as well as renewability, etc. [31,32]. Accordingly, laccase has been considerably highlighted by many researchers in recent years. Laccase has strategic significance to address severe environmental pollution issues [33], and meet the tendency concerning green manufacturing, as well as sustainable development [34]. In addition, laccase has been employed in biosynthesis of biomass pigments and decolorization of synthetic dyestuffs in dyeing industry [27,34,35].

Tea polyphenols are the main component in green tea [36], which also is one of typical substrates for laccase-catalyzed oxidative polymerization [37]. Tea polyphenols would be firstly catalyzed into quinones [32], which could be transformed into theaflavin, since generated quinones were unstable. Theaflavin could be converted into theabrownin via non-enzymatic browning reaction through adding exogenous additive amino acids [38], which could not only further enhance the content of tea pigments, but also could endow aromatic flavor by dyeing fabrics. Therefore, this technology is able to achieve the processes of both dyeing and functional finishing [39].

**Figure 1:**
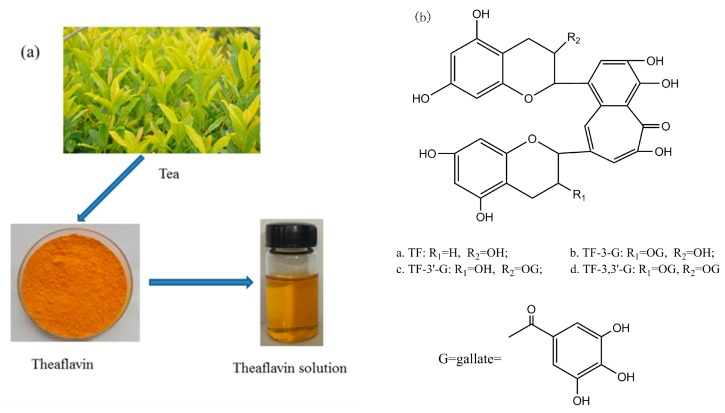
(**a**) Tea leaves, theaflavin, as well as theaflavin solution; (**b**) Chemical structural formula of theaflavin.

Theaflavin is the major component in black tea (Figure 1a), which is the primary oxidation product during the process of tea fermentation [40]. Laccase is able to catalyze precursor tea polyphenols transformed into theaflavin [41], and theaflavin is the mixture. There are four principal substances in theaflavin (Figure 1b), and the chemical structural formula depends on theaflavin (TF), theaflavin-3-gallate (TF-3-G), theaflavin-3′-gallate (TF-3′-G), as well as theaflavin-3,3′-gallate (TF-3,3′-G) [42].

In this study, biocolorant prepared from phenolic compound was achieved, and dyeing of silk and wool fabrics with pigment derived from tea polyphenols was investigated under the influence of pH variation. Accordingly, a novel dyeing method based on pH-induced fixation was established for protein textiles.

## 2. Materials and Methods

### 2.1. Materials

Food-grade colorless tea polyphenols were purchased from Liyuan Food Additives Limited Company of Guangzhou in Guangdong Province of China, which were treated by decolorization processing, and the content of effective substance was 99%. 

The wool fabric (warp density 86 yarns per inch, weft density 51 yarns per inch; weight 132.0 g/m^2^) was purchased from Jiangsu Huaxi Spinning Limited Company (Suzhou, China). The silk fabric (warp density 325 yarns per inch, weft density 34 yarns per inch; weight 75.0 g/m^2^) was bought from FING SILK Limited Company (Hangzhou, China).

Both citric acid and disodium hydrogen phosphate were analytical reagents, and purchased from Tianjin Comio Chemical Reagent Co., Ltd. (Tianjin, China). Laccase (EC1.10.3.2) Denilite II S was bought from Novozymes Corporation (Beijing, China), which was prepared from *Aspergillus* through utilizing submerged fermentation, and the standard enzyme activity was 120 LAMU/g (LAMU= Laccase Units of Modified *Aspergillus*).

### 2.2. Preparation of Biopigment with Laccase

Tea polyphenols (5 g) were added to a buffer solution containing both 0.1 M citric acid and 0.2 M disodium hydrogen phosphate, and the pH value was adjusted to 4.5. Then, 1.0 g (120 LAMU) laccase was placed in that, and volume adjusted to 500 mL. Eventually, all shake flasks were cultivated in an incubator shaker (Shanghai Zhicheng Analytical Instrument Limited Company, Shanghai, China) at 60 °C and 180 rpm.

### 2.3. Dyeing Procedure

#### Dyeing of Protein Fabric under Different pH

The pH value of theaflavin, based on laccase-catalyzed oxidative polymerization for tea polyphenols, was adjusted to 3, 5, 7, 9, as well as 11, respectively, and then silk and wool fabrics were placed in dyeing tanks according to liquor ratio 1:50. Dyeing experiment was carried out in an infrared dyeing equipment (Data color corporation, State of New jersey, USA). The dyeing temperature was 100 °C and soaking time was 60 min, which started from indoor temperature 30 °C with a heating rate of 3 °C/min. At the end of dyeing process, dyed fabrics were washed under running water, and also, were carried out via employing 2 g/L neutral soap flakes at 80 °C for 10 min to wash away residual uncombined pigment from fabrics. After soaping, fabrics were washed with water at 80 °C twice, and washed under running water, followed by drying in a drying oven.

### 2.4. Measurements

#### 2.4.1. Color Characteristics

The CIE L*, a*, b*, C*, h, and *Integ* values, were measured by employing Data color 600 spectrophotometer (Data color corporation, NJ, USA) under photosource D65, 10° visual angle. The measured results were an averaged value from four different locations. The *Integ* value could be calculated according to the following Equation (1):

*Integ* = F(*X*) + F(*Y*) + F(*Z*) (1)

where F(*X*), F(*Y*) as well as F(*Z*) are pseudo tristimulus values.

#### 2.4.2. Color Fastness

The rubbing, soaping, as well as light fastness of dyed fabrics were measured on the basis of ISO 105-C01, ISO 105-X12, as well as ISO 105-B02, respectively.

#### 2.4.3. Breaking Strength

The breaking strength of dyed protein fabrics was measured by the YG065 electronic fabric strength tester (Changzhou No. 1 Textile Equipment Co. Limited, Changzhou, China) according to GB/T 3923.1-2013: Textiles-Tensile properties of fabrics—Part1. The length and width were 100 mm and 25 mm, respectively, and the tensile velocity was 100 mm/min.

## 3. Results and Discussion

### 3.1. Bioconversion of Tea Polyphenols into Dyestuffs

**Figure 2:**
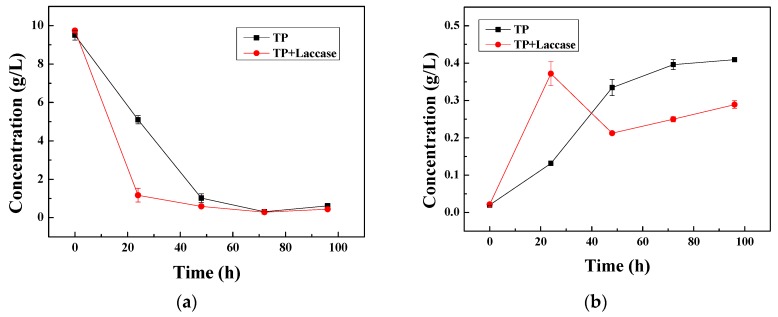
(**a**) Relationship between concentration of tea polyphenols and time; (**b**) Relationship between concentration of theaflavin and time.

The biotransformation process between tea polyphenols and tea pigment was shown in Figure 2a. The content of tea polyphenols declined with reaction time both enzymatic oxidation and non-enzymatic oxidation on the whole. The content of tea polyphenols reduced to a great degree from 12 to 24 h, and the reduction rate of tea polyphenols by non-enzymatic oxidation was 46.30%, and the reduction rate with laccase was 77.99%, and enhanced 41.69% compared to non-enzymatic oxidation during the first 24 h. It could be declared that enzyme activity of laccase reached the maximum, and precursor tea polyphenols could be transformed into theaflavin. The concentration of both tea polyphenols and theaflavin tended to conformity, with the conversion process between the two sides.

The content of theaflavin increased with reaction time as a whole both enzymatic oxidation and non-enzymatic oxidation (Figure 2b). The content of theaflavin increased obviously from 0 to 24 h, and the increasing rate of theaflavin was 74.75% with non-enzymatic oxidation, and it was 94.22% with laccase, during the first 24 h. It could be made clear that tea polyphenols were converted to theaflavin via laccase-catalyzed oxidative polymerization. Theaflavin could be involved in subsequent reactions, and could regenerate into catechin [41,43]. Therefore, the content of theaflavin declined sharply 24 h later. However, the content of theaflavin based on non-enzymatic oxidation increased constantly with reaction time. Accordingly, the process of enzymatic oxidation played a leading role during the whole process. 

**Figure 3:**
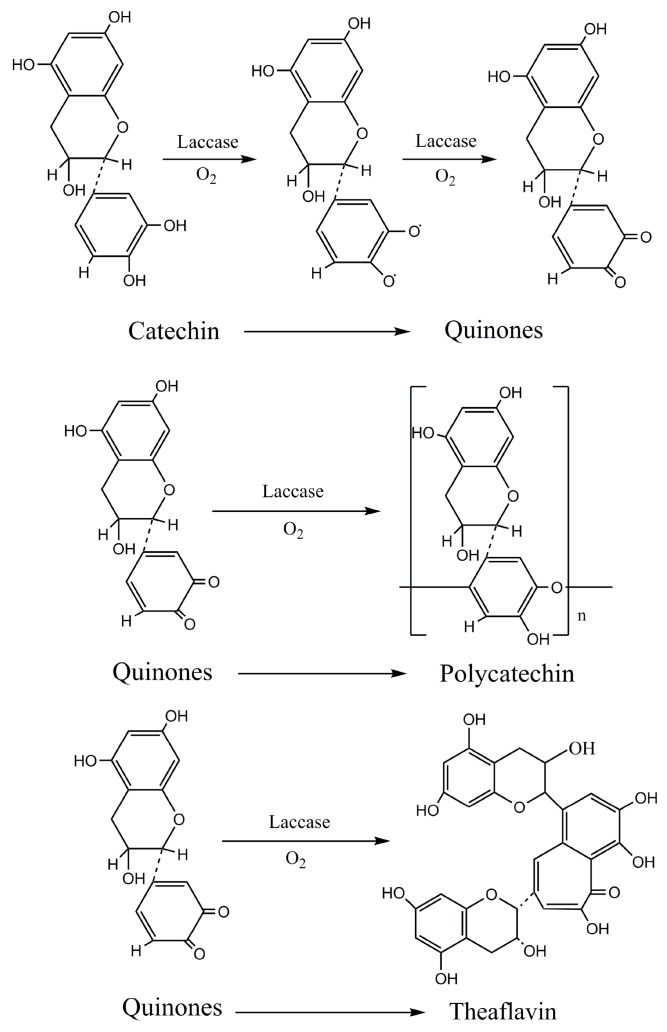
Reaction mechanism of enzymatic oxidation with laccase.

Catechin is the major component in tea polyphenols, which could be firstly catalyzed into active free radicals, and then transformed into quinone intermediates promptly, since free radicals are considerably unstable [29]. Quinone intermediates could carry out subsequent polymerization reactions by means of high reaction activity (Figure 3) [38,40], and catechin monomer connected mutually in the form of ether bonds in the structure of reaction product, polycatechin [32].

**Figure 4:**
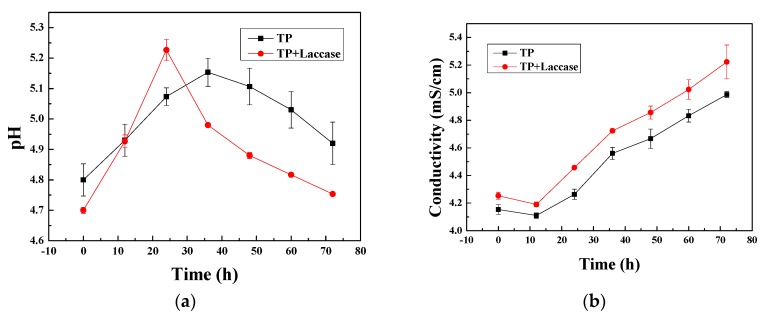
(**a**) Change between pH value and reaction time; (**b**) Change between conductivity and reaction time.

Tea polyphenols possessed weak-acid properties, since phenolic hydroxyl groups could ionize hydrogen ions in aqueous solution. The pH value of reaction solution presented increase at the early stage, and then decreased both in enzymatic oxidation and non-enzymatic oxidation (Figure 4a). The pH value of reaction liquid achieved the maximum at 24 h, based on laccase-catalyzed oxidative polymerization, and then reduced over the next few hours. The possible reason was that theaflavin could be regenerated into catechin by both enzymatic oxidation and non-enzymatic oxidation.

The conductivity of reaction solution presented a decrease at first, and then increased in both enzymatic oxidation and non-enzymatic oxidation (Figure 4b). The phenolic hydroxyl groups in tea polyphenols could ionize hydrogen ion sin aqueous solution, and could firstly be formed into active free radicals, and then transformed into quinone intermediates promptly, because free radicals were considerably unstable. Therefore, the conductivity of reaction solution would be firstly reduced. Quinones would be converted to theaflavin firstly, and then further transformed into thearubigins and even theabrownin via oxidative polymerization during subsequent reactions [38]. Accordingly, the conductivity of reaction solution would be increased steadily over the upcoming few hours.

### 3.2. Dyeing of Protein Fabric with Tea Biomass Pigments

The apparent color and color parameters of dyed silk fabrics were shown in Table 1 and Table 2. The *Integ* value was the maximum under the condition of pH 3, and all of the values *Integ*, a*, b*, and C* were positive, and decreased with the increase of pH value. It could be explained that the colored lights both red and yellow, as well as saturability of dyed fabrics, were reduced. 

**Figure 5:**
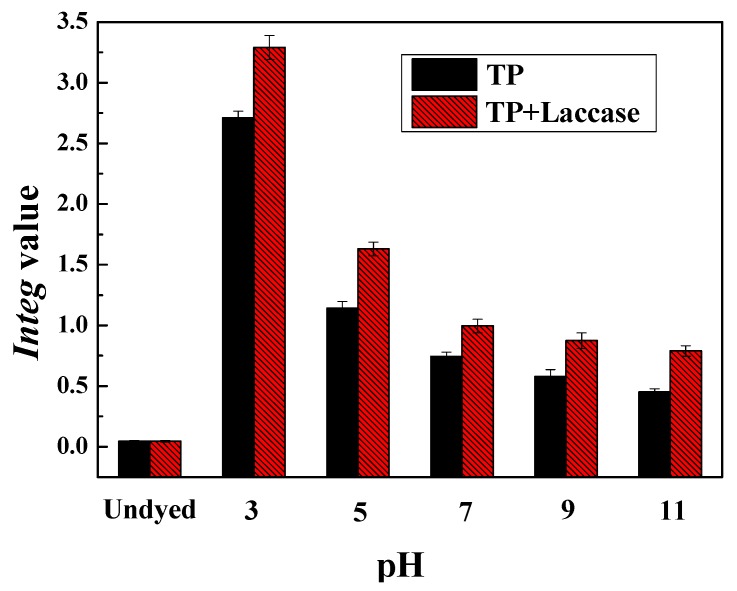
Effect of pH value on *Integ* value of dyed silk fabrics.

The *Integ* value of dyed silk fabrics decreased with the increase of pH value in reaction solution from acidity to alkalinity, in both enzymatic oxidation and non-enzymatic oxidation, and the *Integ* value with laccase was greater compared to the control (Figure 5). In addition, the *Integ* value of dyed silk fabrics was the maximum when dye bath pH was 3. It was declared that theaflavin was relatively steady under acidic conditions, and could be converted to macromolecular thearubigins and even theabrownin through oxidative polymerization under the condition of alkalinity. Micromolecular theaflavin could diffuse into silk fabric, while macromolecular thearubigins and even theabrownin could not penetrate into that, since the structure of silk was relatively tight compared to wool fabric.

**Figure 6:**
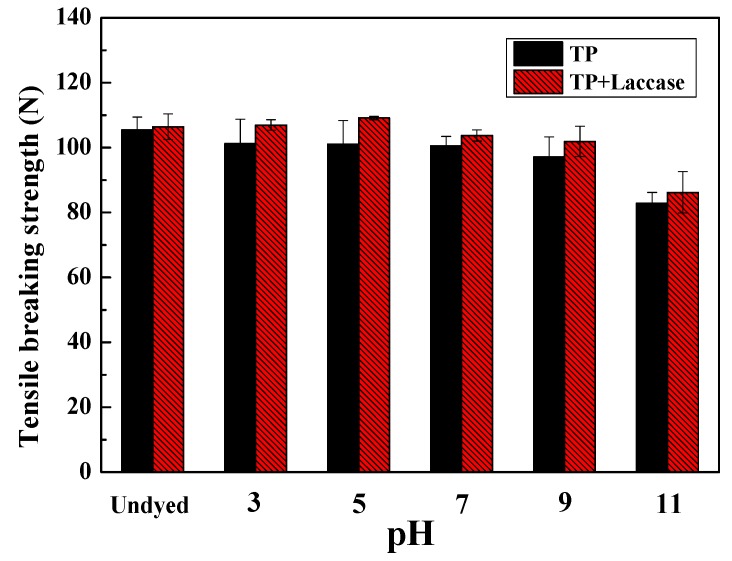
Effect of pH value on breaking strength of dyed silk fabrics.

The breaking strength of dyed silk fabrics reduced dramatically when dye bath pH was 11, and tensile breaking force dyed by TP + laccase was greater compared to the control (Figure 6). It could be explained that silk did not have alkali-resistance properties, and would hydrolyze to amino acids under alkaline conditions. Therefore, the binding forces between the molecules would be destroyed. Additionally, the van der Waals force of TP + laccase was bigger than the counterpart, possibly since the molecular weight of tea pigments was relatively large.

It was obvious that the color strength of dyed wool fabrics was the maximum when pH value was 3 according to Table 3 and Table 4, and the L* value of dyed fabrics was increased with the increase of pH value. Moreover, the hue angle h value of dyed wool was less than 90 degrees, and the color of dyed fabrics presented yellowish-brown, accordingly. On another level, the effect of pH value on color was related to the optimal pH of laccase.

**Figure 7:**
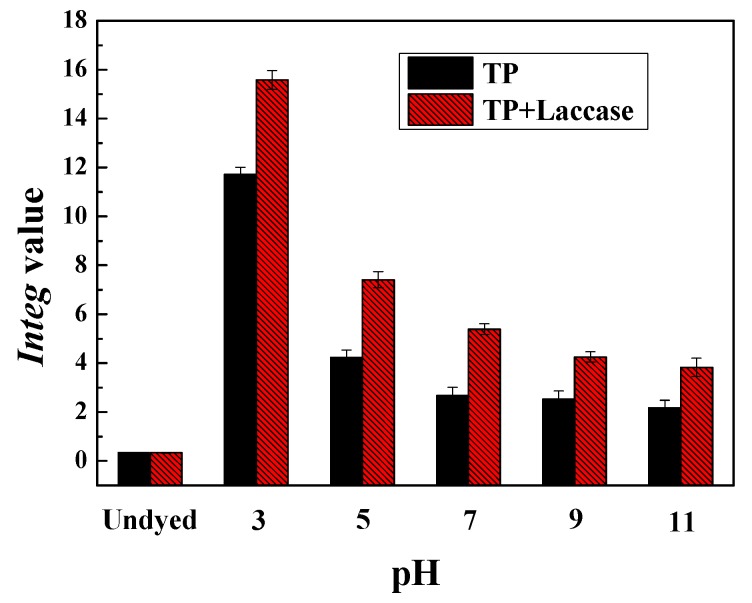
Effect of pH value on *Integ* value of dyed wool fabrics.

**Figure 8:**
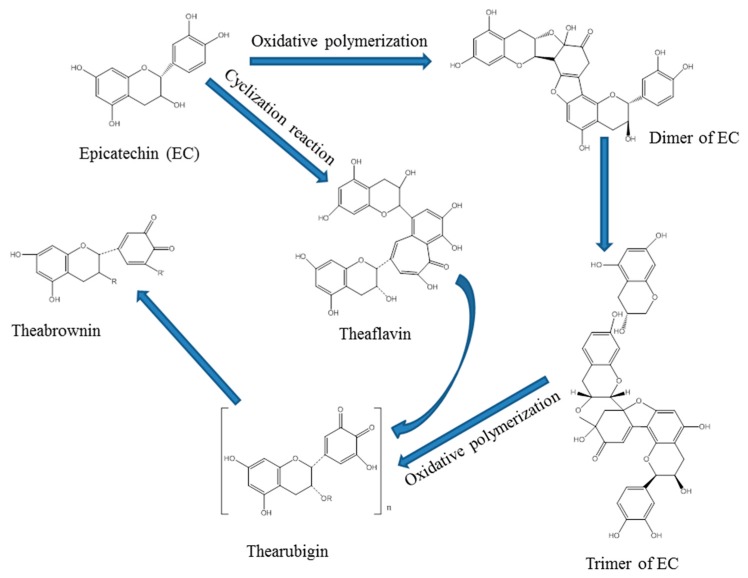
Major conversion pathways concerning non-enzymatic oxidation between catechin and tea pigments.

The *Integ* value of dyed wool fabrics with non-enzymatic oxidation was greater compared to their counterpart under acidic conditions. However, experimental results indicated that conclusion was contrary compared to acidic conditions (Figure 7). The major component of tea pigments in reaction solution was micromolecular theaflavin under acidic conditions. Therefore, theaflavin could easily diffuse into wool fabric under relatively high temperature conditions, and the content of theaflavin in dye liquor with non-enzymatic oxidation was even higher than that. Additionally, theaflavin could be transformed into macromolecular thearubigins, and even theabrownin, under neutral and alkaline conditions (Figure 8) [39], under which it was hard to penetrate into wool fabric. Hence, the *Integ* value of dyed wool fabrics was lower compared to acidic conditions.

**Figure 9:**
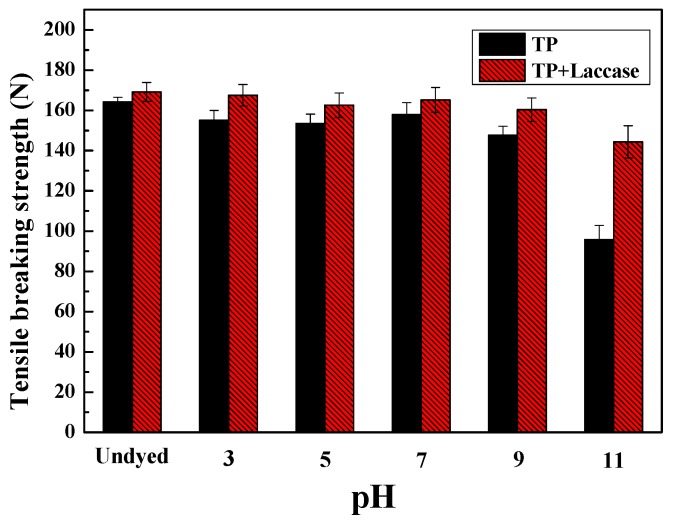
Effect of pH value on breaking strength of dyed wool fabrics.

The breaking strength of dyed wool fabrics reduced sharply when dye bath pH was 11, and tensile breaking force dyed by TP + laccase was greater compared to the control (Figure 9). It could be accounted for that wool did not have alkali-resistance properties, and would hydrolyze to amino acids under alkaline conditions. Therefore, the binding forces between the molecules would be destroyed. Furthermore, wool possesses scale layer structure, and is heavier compared to silk fabric. Accordingly, the mechanical properties were better than silk. 

The adsorption mechanism between tea pigments and protein fabric could meet the adsorption isotherm of Langmuir model below the isoelectric point [44]. However, it conformed to the adsorption isotherm of Freundlich model above the isoelectric point, and the diffusion model accorded with pore diffusion model, since both silk and wool fabrics were hydrophilic fibers. The combination mode between tea pigments and wool fabric was mainly ionic bonds below the isoelectric point, but the *Integ* value of wool fabrics was greater compared to dyed silk fabric. The possible reason was that the content of amino was almost equal to the content of carboxyl in wool fiber, while the quantity of amino was less than carboxyl in silk fiber. On another level, wool is a porous material with capillary effect; hydrotropic substances were easily adsorbed into fiber gaps or surfaces, accordingly. In addition, tea pigments prepared from tea polyphenols were water-soluble, and the quantity as well as variety of hydrophilic groups much more compared to silk. Additionally, tea polyphenols could possess negative charges under conditions of acidity, and protein fiber could have positive charges below the isoelectric point. Accordingly, the combination mode between tea polyphenols and wool fabric, including intermolecular force as well as electrostatic force, and the dyeing property of wool fabric was the greatest among all dyed fabrics.

### 3.3. Fixation under Different Acid and Base Conditions

Color fastness (rubbing, washing, as well as light fastness) of dyed silk under the different condition of acidity and alkalinity are shown in Table 5 and Table 6. The fastness, both rubbing and washing, were measured according to five level standard, while light fastness was tested in the light of eight level standard. Accordingly, the rubbing and washing fastness were considerably admirable, and could meet application requirements. 

Color fastness (rubbing, washing, as well as light fastness) of dyed wool, under the different conditions of acidity and alkalinity, is shown in Table 7 and Table 8. The phenolic hydroxyl group in theaflavin and tea polyphenols would ionize hydrogen ions, and could have negative oxygen ions under the conditions of acidity. Additionally, protein fiber could ionize ammonium ion below the isoelectric point, and the combination mode between fiber and colorant was electrostatic force. Therefore, the fixation rate of dyed fabrics was better compared to alkalinity, since the binding force was intermolecular force under alkaline conditions.

Micromolecular theaflavin and tea polyphenols would be absorbed on the surface of fabric under acidic conditions, and then could diffuse into fiber with the constant rise of temperature. Micromolecular substances would be oxidized to macromolecular thearubigins and even theabrownin under the conditions of both oxygen and high temperature during the process of dyeing. Therefore, color fastness among rubbing, washing, and light fastness were better under the conditions of acidity compared to alkalinity.

Theaflavin prepared from tea polyphenols was able to carry out reversible response color change under different acid and base conditions. Theaflavin was stable under acidic conditions, while it would be transformed into thearubigins and even theabrownin under the conditions of either alkalinity or high temperature. Theaflavin is a micromolecular substance, which would be firstly absorbed on the surface of fabric, then could diffuse into fibers and be oxidized into macromolecular tea pigments via oxidation polymerization. Accordingly, the degree of fixation was better under acidic conditions compared to alkalinity.

Compared with synthetic dyestuffs, biocolorant was not admirable to combine with fiber [45]. Accordingly, both exhaustion rate and color fastness were low during the process of dyeing with biocolorant, generally. Mordants were applied in traditional technologies, concerning dyeing with natural pigments to improve dyeing properties [46,47]. However, most mordants were poisonous and forbidden in eco-textiles. In this research, the dyeing of biocolorant derived from tea polyphenols was achieved, and no mordant was employed to enhance the fixation. Therefore, the dyed fabrics are able to reach the standard of ecological textile.

## 4. Conclusions

The reduction rate of tea polyphenols with non-enzymatic oxidation was 46.30%, and the increasing rate of theaflavin was 84.75% during the first 24 h. Additionally, the reduction rate of tea polyphenols with laccase was 77.99%, and the increasing rate of theaflavin was 94.22% during the first 24 h. Accordingly, the reduction rate of tea polyphenols and the increasing rate of theaflavin were enhanced 41.69% and 19.47%, respectively, compared to non-enzymatic oxidation.

The preparation of theaflavin from tea polyphenols with laccase was carried out, and dyeing of protein fabric was achieved from acidity to alkalinity, in this investigation. Experimental results demonstrated dyeing properties were better under acidic conditions compared to alkalinity, and both dyeing property and fixation rate were the best when pH value was 3. In addition, the dyeing property of wool fabric was better than silk when dyed by identical dye liquor.

Nowadays, natural products especially derived from plants, are gaining popularity around the globe for their application in textiles, by virtue of abundant availability, biocompatibility, low toxicity, compatibility with green approaches, and eco-friendly nature. Tea is the predominant plant resource in China, and large amounts of tea stem and other waste will be produced during the processing period, which provides rich raw material for the extraction of natural functional substances.

## Figures and Tables

**Table 1:** The color parameters of dyed silk fabrics with tea polyphenols.

Dye Liquor pH	Undyed Silk	3	5	7	9	11
Apparent color	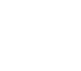	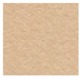	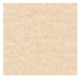	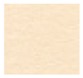	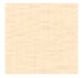	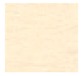
*Integ* value	0.05	2.71	1.14	0.76	0.58	0.47
L*	94.51	68.31	76.78	81.51	82.80	83.83
a*	0.10	5.35	3.63	2.65	2.62	2.46
b*	2.79	19.69	13.24	13.10	11.77	11.66
C*	2.80	18.48	13.59	13.20	12.82	11.95
h	88.05	73.18	74.52	77.36	78.21	78.69

**Table 2:** The color parameters of dyed silk fabrics based on laccase-catalyzed oxidative polymerization for tea polyphenols.

Dye Liquor pH	Undyed Silk	3	5	7	9	11
Apparent color	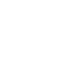	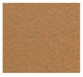	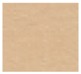	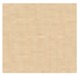	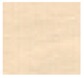	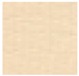
*Integ* value	0.05	3.27	1.65	1.02	0.86	0.75
L*	94.51	67.29	73.00	78.31	79.25	81.05
a*	0.10	5.63	3.69	2.81	2.63	2.45
b*	2.79	18.79	15.84	15.37	14.66	13.87
C*	2.80	19.61	16.26	15.09	14.74	14.09
h	88.05	73.32	76.87	79.00	79.83	79.99

**Table 3:** The color parameters of dyed wool fabrics with tea polyphenols.

Dye Liquor pH	Undyed Wool	3	5	7	9	11
Apparent color	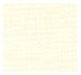	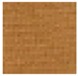	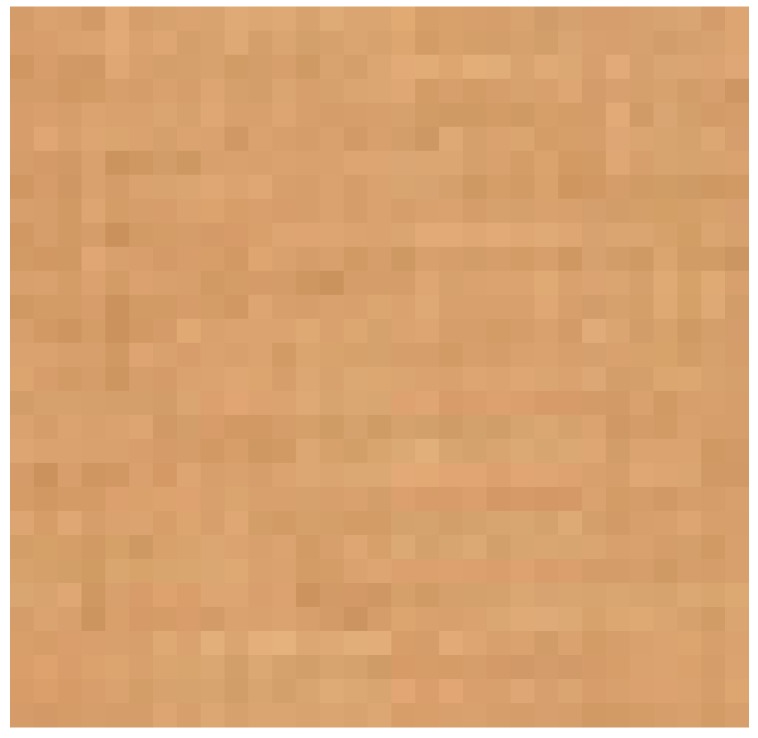	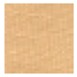	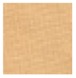	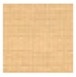
*Integ* value	0.35	11.73	4.24	2.67	2.52	2.17
L*	87.85	48.03	62.98	68.15	69.35	70.75
a*	0.40	10.46	7.79	6.57	6.19	5.78
b*	13.02	27.01	24.39	22.69	21.89	20.61
C*	13.03	28.96	25.60	23.54	23.18	22.65
h	91.76	68.84	72.30	73.80	74.74	75.21

**Table 4:** The color parameters of dyed wool fabrics based on laccase-catalyzed oxidative polymerization for tea polyphenols.

Dye Liquor pH	Undyed Wool	3	5	7	9	11
Apparent color	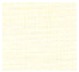	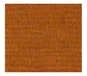	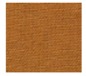	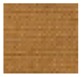	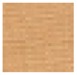	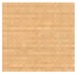
*Integ* value	0.35	15.82	7.92	5.52	4.46	3.99
L*	87.85	47.76	52.04	56.28	66.58	67.92
a*	0.40	9.48	7.61	7.30	6.81	6.65
b*	13.02	25.53	24.42	24.33	24.10	23.49
C*	13.03	27.23	25.58	25.27	25.18	24.41
h	91.76	69.63	72.70	73.16	74.20	74.37

**Table 5:** The color fastness of dyed silk fabrics under the conditions of different acidity and alkalinity.

Dye Liquor pH	Rubbing Fastness		Washing Fastness			Light Fastness
	Dry	Wet	CC	SC	SW	
3	5	4–5	5	5	5	4–5
5	5	4–5	4–5	5	5	4–5
7	4–5	4–5	4–5	4–5	4–5	4
9	4–5	4	4	4	4	4
11	4–5	4	4	4	4	4

Color change (CC), staining on cotton fabric (SC), staining on wool fabric (SW).

**Table 6:** The color fastness of dyed silk fabrics based on catalytic oxidation with laccase.

Dye Liquor pH	Rubbing Fastness		Washing Fastness			Light Fastness
	Dry	Wet	CC	SC	SW	
3	5	5	5	5	5	5
5	5	5	5	5	5	5
7	5	4–5	5	5	5	4–5
9	4–5	4	4–5	4–5	4–5	4–5
11	4–5	4	4–5	4–5	4–5	4–5

Color change (CC), staining on cotton fabric (SC), staining on wool fabric (SW).

**Table 7:** The color fastness of dyed wool fabrics under different acid and base conditions.

Dye Liquor pH	Rubbing Fastness		Washing Fastness			Light Fastness
	Dry	Wet	CC	SC	SW	
3	5	4–5	4–5	4–5	4–5	4–5
5	5	4–5	4–5	4–5	4–5	4–5
7	5	4	4–5	4–5	4–5	4
9	4–5	4	4	4	4	4
11	4–5	4	4	4	4	4

Color change (CC), staining on cotton fabric (SC), staining on wool fabric (SW).

**Table 8:** The color fastness of dyed wool fabrics based on catalytic oxidation with laccase.

Dye Liquor pH	Rubbing Fastness		Washing Fastness			Light Fastness
	Dry	Wet	CC	SC	SW	
3	5	5	5	5	5	5
5	5	5	5	5	5	5
7	5	4–5	4–5	5	5	4–5
9	4–5	4–5	4–5	4–5	4–5	4–5
11	4–5	4–5	4–5	4–5	4–5	4–5

Color change (CC), staining on cotton fabric (SC), staining on wool fabric (SW).
